# Recurrent Ameloblastoma of the Mandibular Ramus Resected Using a Modified High Perimandibular Approach: A Case Report

**DOI:** 10.1155/crid/3560799

**Published:** 2026-04-07

**Authors:** Atsushi Uesugi, Kumiko Kamada, Keiko Kudoh, Naito Kurio

**Affiliations:** ^1^ Department of Oral Surgery, Institute of Biomedical Sciences, Tokushima University Graduate School, Tokushima, Japan, tokushima-u.ac.jp

## Abstract

Ameloblastoma typically lacks a well‐defined capsule and exhibits locally invasive growth, making them prone to recurrence. On the other hand, a subtype known as the unicystic type is generally well circumscribed and less invasive compared with conventional solid/multicystic variants, but recurrence may occur in rare cases. Therefore, after tumor removal, it is essential to resect or curette the adjacent bone surface that may have been infiltrated. The high perimandibular approach is a surgical technique originally developed for treating condylar fractures. It involves a 4–5 cm skin incision placed 5 mm below the lower border of the mandible, followed by incision of the platysma and masseter muscles above the course of the mandibular branch of the facial nerve, thereby protecting the nerve. Compared with other surgical approaches, this method provides a wide operative field and superior esthetic outcomes, as postoperative scars are less conspicuous. In this report, we describe a case in which this approach was adapted for resecting a recurrent ameloblastoma of the mandibular ramus. The patient was a 58‐year‐old man who presented to our department in May 2016 with a lesion extending from the right mandibular body to the mandibular ramus. A diagnosis of ameloblastoma unicystic type was established, and fenestration procedures were performed in May 2017. However, further treatment was discontinued at the patient’s request. The tumor subsequently recurred on the lateral surface of the right mandibular ramus, measuring 28 mm × 14 mm. In May 2022, the lesion was resected using the extended high perimandibular approach: an extended incision of 10 cm, twice the usual length, provided sufficient surgical exposure to allow for safe and effective complete tumor removal. Postoperatively, no facial nerve paralysis occurred, and no recurrence was observed during 3 years of follow‐up. Esthetic outcomes were satisfactory. In conclusion, the extended high perimandibular approach may be a useful surgical option for managing recurrent ameloblastoma on the lateral surface of the mandibular ramus.

## 1. Introduction

Ameloblastomas often lack a distinct capsule and exhibit invasive growth, making them prone to recurrence. On the other hand, a subtype known as the unicystic type is generally well circumscribed and less invasive compared with conventional solid/multicystic variants, but recurrence may occur in rare cases. Therefore, after surgical removal, it is essential to remove a layer of adjacent bone that may have been infiltrated by the tumor. The high perimandibular approach, a surgical technique commonly used for treating condylar fractures, was first described by Wilk et al. [1]. This method involves a 4–5 cm skin incision placed 5 mm below the lower border of the mandible, with an incision of the platysma and masseter muscles above the course of the mandibular branch of the facial nerve to access the condylar process. The advantages of this approach include easy access to a wide surgical field, clear visualization of the condylar region, minimal injury risk to the mandibular branch of the facial nerve, and inconspicuous scarring hidden in the shadow of the mandibular border. In this report, we describe the effectiveness of an extended high perimandibular approach, in which the incision length was doubled to 10 cm, for resecting a 28 mm × 14 mm recurrent ameloblastoma located on the lateral surface of the mandibular ramus.

## 2. Case Presentation

A 58‐year‐old man presented to our department in May 2016 for further evaluation of a radiographic finding of a lesion extending from the right mandibular body to the mandibular ramus. Computed tomography revealed a cystic lesion extending from the coronoid process of the right mandibular ramus to the area corresponding to 6┐, which included the crown of impacted tooth 8┐, and an odontogenic keratocyst or ameloblastoma was suspected. His medical history included schizophrenia and hyperlipidemia, both managed with medication. A biopsy of the right mandible confirmed a histopathological diagnosis of ameloblastoma. Fenestration procedures were performed in May 2017; however, further treatment was discontinued at the patient’s request. In March 2022, recurrence was detected, with a lesion measuring 28 mm × 14 mm on the lateral surface of the right mandibular ramus, extending from the anterior to posterior margins (Figure 1).

Figure 1Recurrence of the lesion observed from the anterior to posterior margins of the lateral surface of the right mandibular ramus. A preoperative computed tomography image is shown. Arrow indicates recurrent lesion on the anterior margin of the mandibular ramus. Arrowhead indicates recurrent lesion on the posterior margin of the mandibular ramus. (a) Sagittal section. (b) Coronal section. (c) Three‐dimension.

**Figure:**
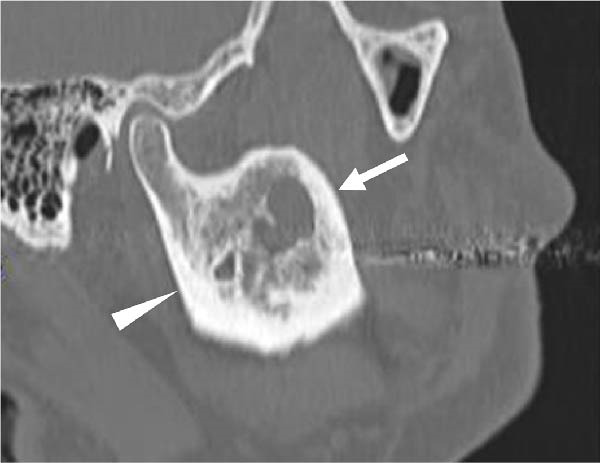
(a)

**Figure:**
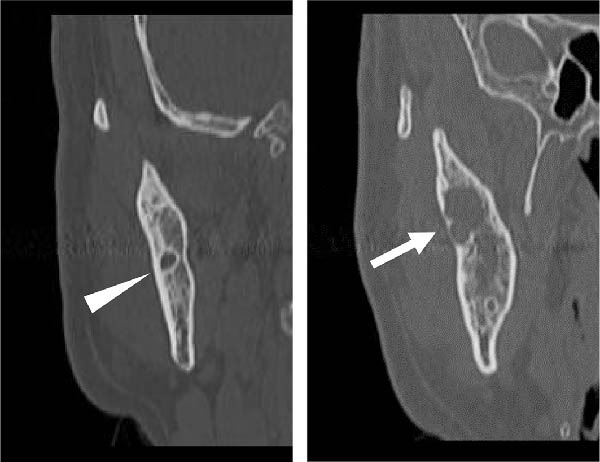
(b)

**Figure:**
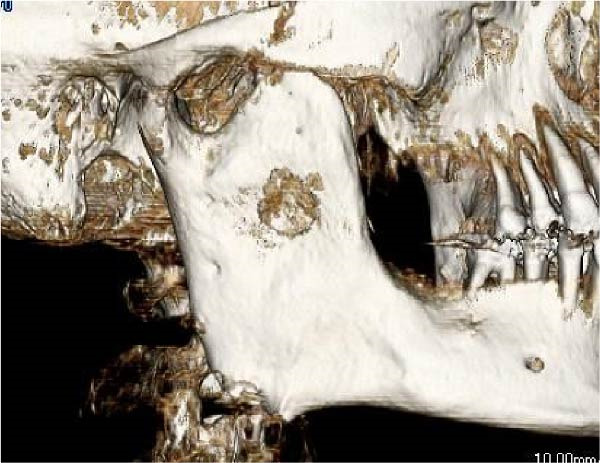
(c)

Accordingly, in May 2022, surgical resection was planned using a high perimandibular approach. A 10‐cm skin incision, twice the usual length, was made 5 mm below the lower border of the right mandible (Figure 2a). The platysma muscle was exposed, and the overlying skin was undermined and elevated superiorly (Figure 2b). The platysma was then incised 1–2 cm above the lower border of the mandible, exposing the underlying masseteric fascia. The buccal branch of the facial nerve was identified and retracted upward for protection (Figure 2c). The masseter muscle was dissected subperiosteally, exposing the lesion on the lateral surface of the mandibular ramus (Figure 2d). Subsequently, the tumor was resected together with portions of the periosteum and masseter attached to the specimen, and the adjacent bone surface was burred to eliminate possible microscopic invasion. The surface of the bone was removed in layers until the rough bone surface was smooth (Figure 2e). The anterior and posterior tumor cavities were connected to form a single surgical cavity (Figure 2f). Adequate visibility was maintained throughout the procedure, and the wound was closed primarily. Histopathological examination revealed a unicystic type ameloblastoma (Figure 3).

Figure 2Surgical procedure. (a) A 10‐cm skin incision is made 5 mm below the lower edge of the right mandible, twice the usual length. (b) The platysma muscle is exposed, and the skin is undermined and dissected upward. (c) The buccal branch of the facial nerve is exposed and pulled upward. (d) The exposed masseter muscle is dissected below the periosteum, exposing the lesion on the lateral aspect of the mandibular ramus. (e) The lesion is removed, and the bone surface adjacent to the lesion is scraped with a burr. (f) The anterior and posterior wounds are joined to form a single cavity.

**Figure:**
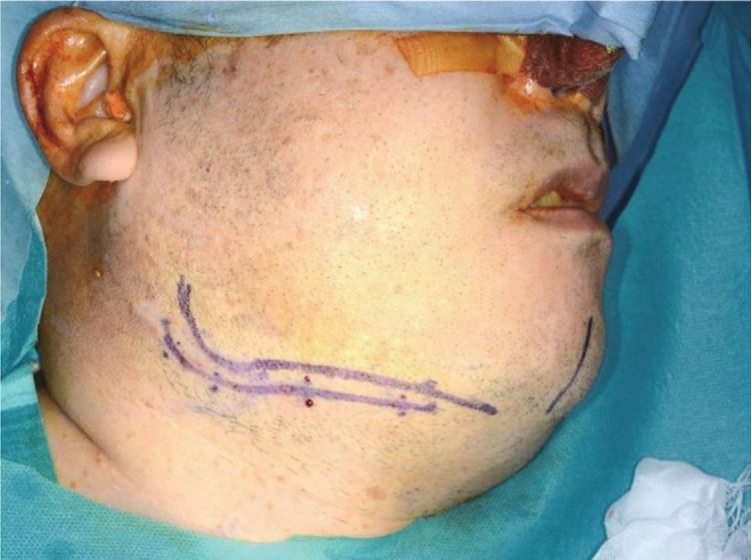
(a)

**Figure:**
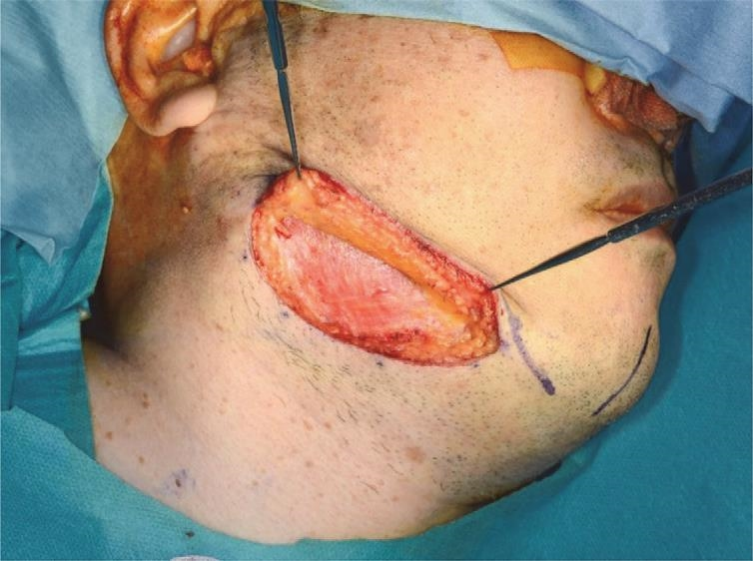
(b)

**Figure:**
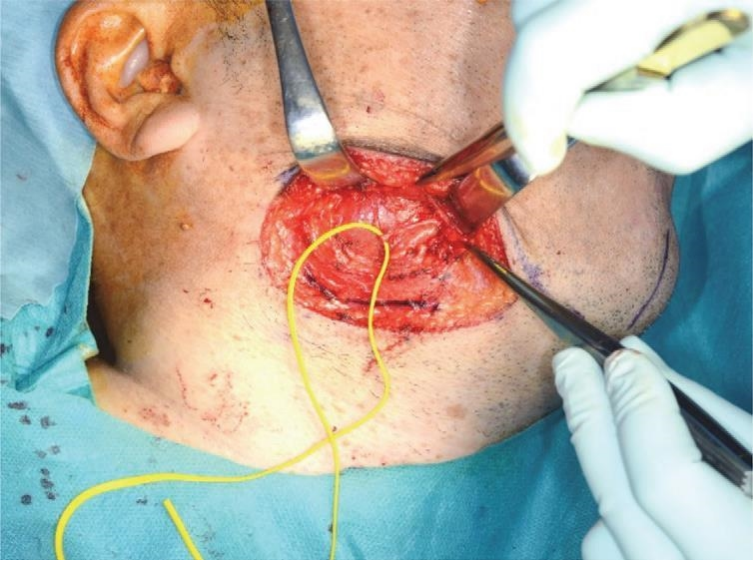
(c)

**Figure:**
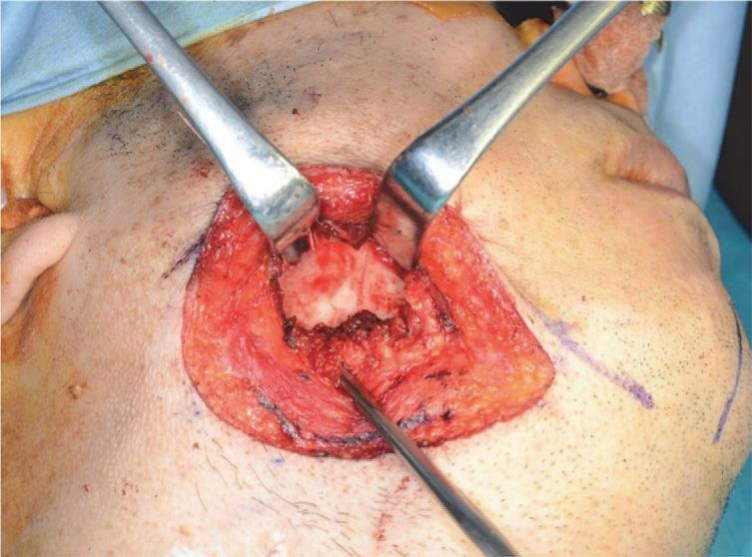
(d)

**Figure:**
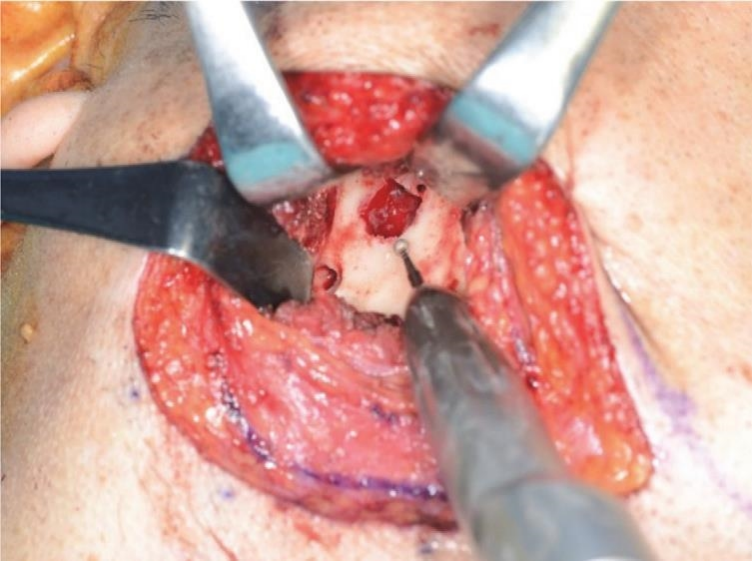
(e)

**Figure:**
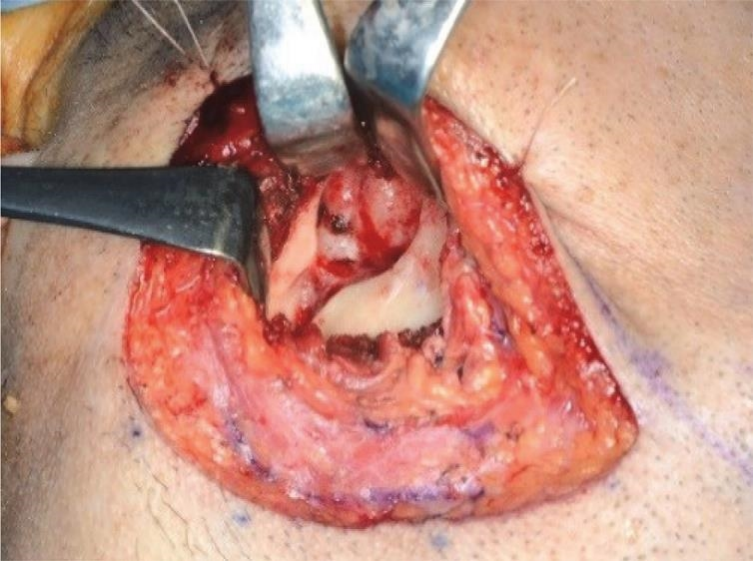
(f)

Figure 3(a) Anterior surgical specimen, 12 mm × 12 mm. (b) Posterior surgical specimen, 6 mm × 4 mm. Histopathological examination confirmed unicystic type ameloblastoma in both areas. Scale bar = 500 µm.

**Figure:**
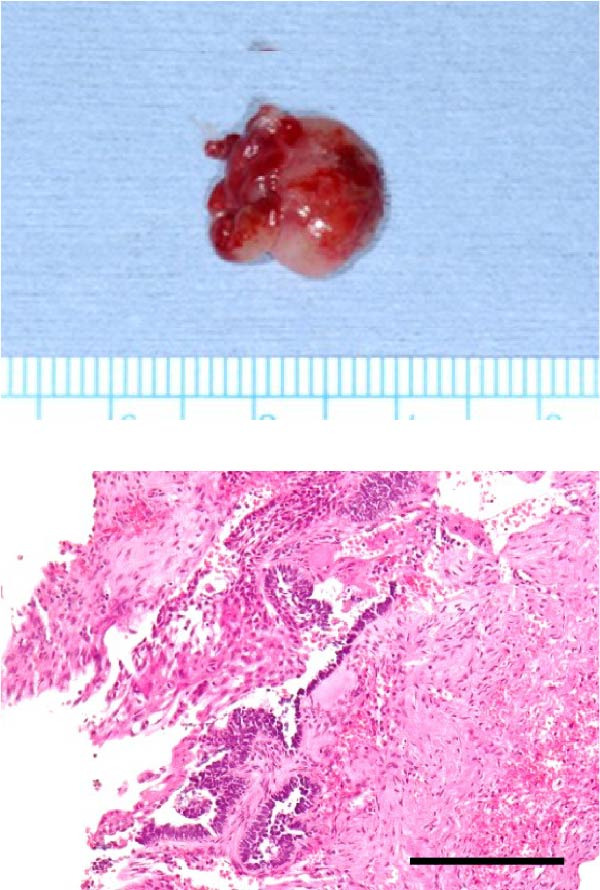
(a)

**Figure:**
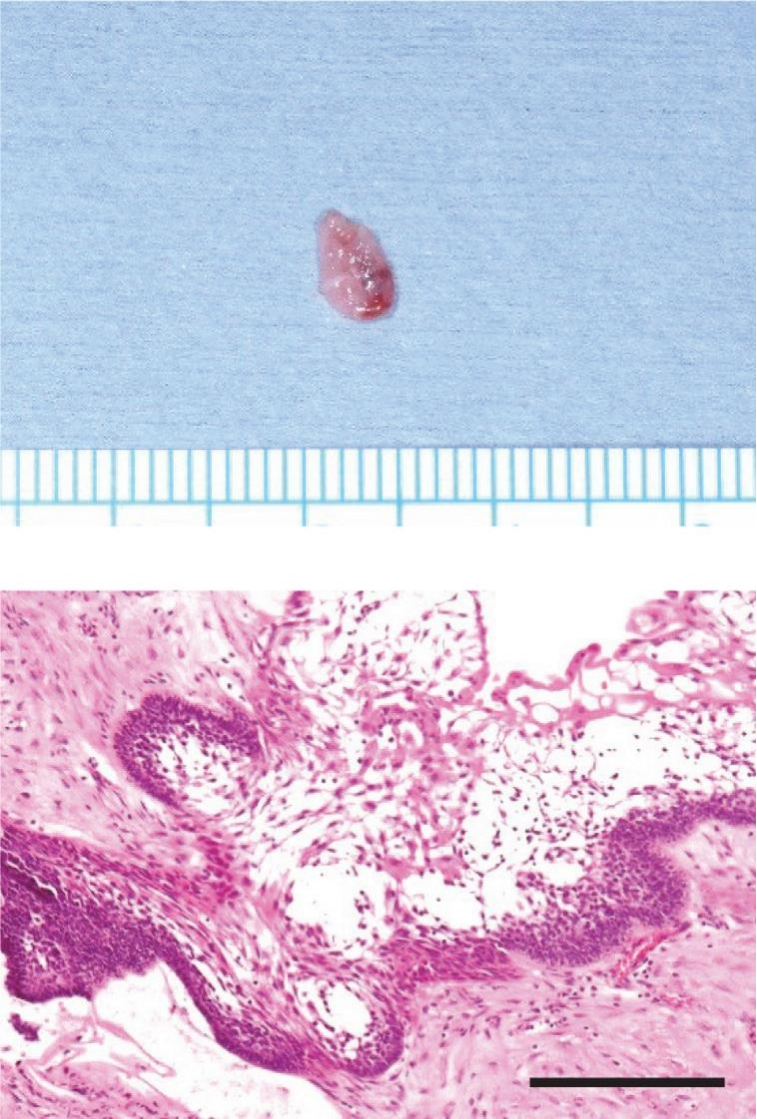
(b)

**Figure 5:**
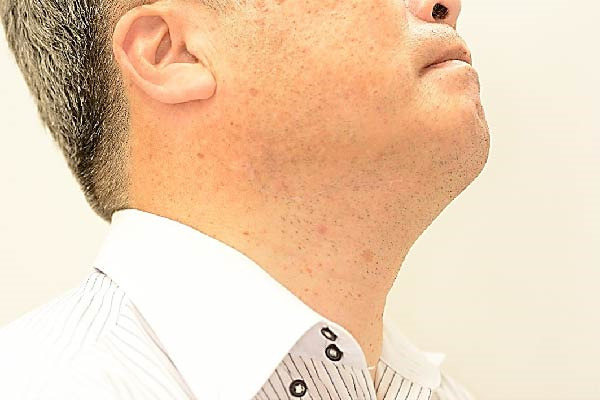
Although the skin incision length was doubled, the patient’s postoperative course was favorable, with no noticeable scar formation.

The postoperative course was uneventful. No facial nerve paralysis occurred, and no recurrence was observed during the 3‐year follow‐up (Figure 4). Although the skin incision length was doubled, the resulting scar was inconspicuous (Figure 5), and the esthetic outcome was satisfactory.

Figure 4Computed tomography image obtained 3 years after surgery showing no evidence of lesion recurrence. (a) Sagittal section. (b) Coronal section. (c) Three‐dimension.

**Figure:**
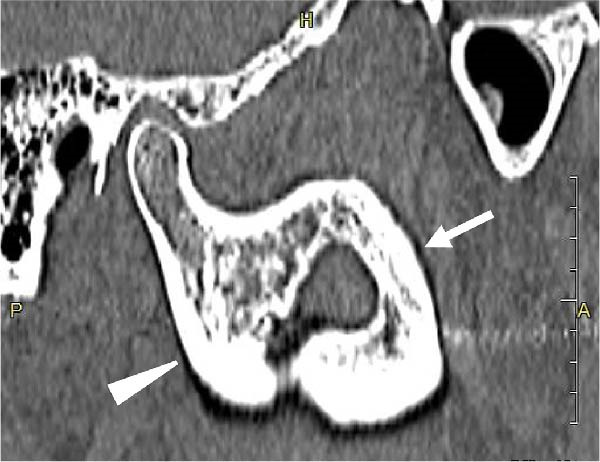
(a)

**Figure:**
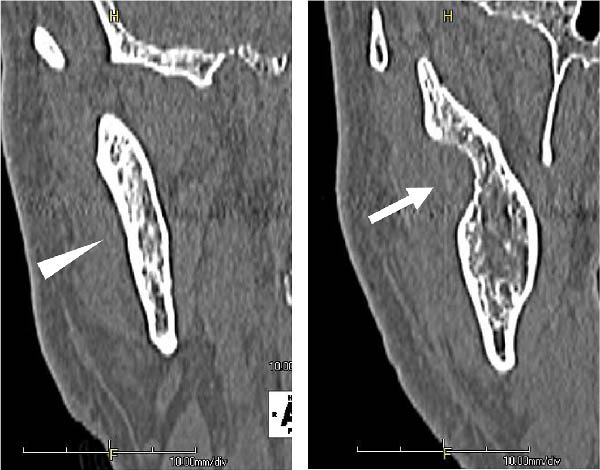
(b)

**Figure:**
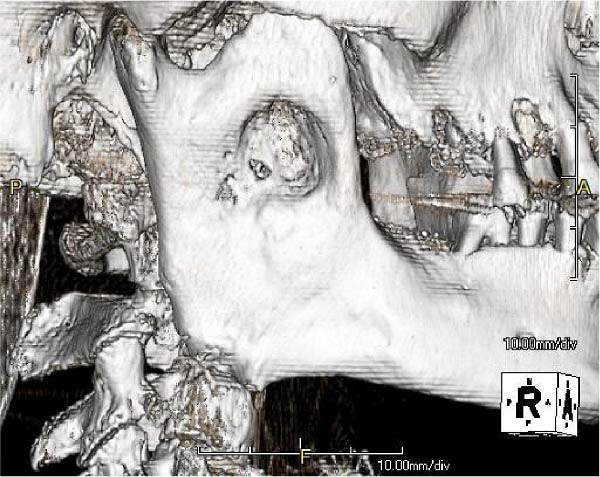
(c)

## 3. Discussion

As ameloblastomas are prone to recurrence, it is essential to remove the adjacent bone surface that may have been infiltrated after removing the tumor. However, when the tumor occurs on the lateral surface of the mandibular ramus, surgical access is difficult and requires a creative approach. In the case we described, we used an extended high perimandibular approach, which is commonly used for treating condylar fractures.

For mandibular condylar fractures, various surgical techniques have been described to balance maneuverability, facial nerve preservation, and esthetic outcomes. The submandibular and retromandibular approaches are commonly used, and in 1997, Wilk et al. [1] introduced the high perimandibular approach as an alternative. This method involves a 4–5 cm incision placed 5 mm below the mandibular border, with dissection of the platysma and masseter muscles above the mandibular branch of the facial nerve, thereby protecting the nerve and shortening the distance to the operative site.

For resecting ameloblastomas, the intraoral approach offers advantages in terms of esthetics and avoidance of facial nerve paralysis; however, it has certain limitations. First, it provides poor access to lesions located on the lateral surface of the mandibular ramus or posterior to the mandibular canal. Second, the restricted surgical field makes complete resection difficult. Considering these limitations, we applied the high perimandibular approach to a recurrent ameloblastoma of the mandibular ramus. In contrast to the standard incision, which measures 4–5 cm, including the mandibular angle, we extended the incision to 10 cm in our case to secure a wide surgical field. At this level, the distance to the lateral ramus is shorter than that in the submandibular approach, and the scar remains hidden beneath the mandibular border. The skin flap was elevated superficial to the platysma, after which the platysma was incised to expose the masseteric fascia. The mandibular branch of the facial nerve was not encountered, and the buccal branch—reported to lie on the masseter fascia in about 40% of cases [2]—was preserved by gentle retraction. The masseter muscle was divided subperiosteally, providing exposure of the lateral ramus. This technique requires division of the masseter; however, the risk of postoperative trismus is low [2], and none occurred in our case.

Previous reports have described the high perimandibular approach for venous malformation [3], hemangioma [4], and osteoma [5] but not for ameloblastoma (Table 1). To our knowledge, this is the first report of its application to a recurrent ameloblastoma of the mandibular ramus, which, in our case, was also the largest lesion removed to date. Extending the incision to 10 cm allowed clear visualization of both the anterior and posterior borders, ensuring complete resection. Notably, despite doubling the incision length, esthetic outcomes were favorable.

**Table 1:** Literature reports on the application of the high perimandibular approach to lesions other than condylar fractures.

S. number	Author	Year	Age	Sex	Diagnosis	Location	Lesion size	Skin incision	Facial palsy	Trismus	Esthetic issues	Recurrence
1	Sukedai et al. [3]	2021	46	F	Venous malformations	Intramasseter	2 cm	5 cm	None	None	None	None p.o 12 months
2	Ishizuka et al. [4]	2022	56	F	Hemangiomas	Intramasseter	10 mm	4 cm	None	None	None	None p.o 2 years
3	Ishizuka et al. [4]	2022	70	F	Hemangiomas	Intramasseter	10 mm	4 cm	None	None	None	None p.o 1 years
4	Sekiguchi et al. [5]	2025	63	F	Osteoma	Subcondylar region	Not specified	Not specified	None	None	None	None p.o 2 years
5	This case	—	58	M	Ameloblastoma	Mandibular ramus	28 mm × 14 mm	10 cm	None	None	None	None p.o 3 years

In conclusion, an extended high perimandibular approach, with the skin incision length doubled, was used to resect a recurrent ameloblastoma on the lateral surface of the mandibular ramus. Postoperatively, no facial nerve paralysis occurred, no recurrence has been observed during 3 years of follow‐up, and the surgical scar remained inconspicuous. Our case suggests that the high perimandibular approach is an effective and esthetically favorable option for tumors located on the lateral surface of the mandibular ramus. In cases of ameloblastoma, where recurrence is common, an extended incision provides sufficient exposure for safe resection and may help reduce recurrence risk.

## Funding

No funding was received for this manuscript.

## Consent

Consent for publication was obtained from the participants.

## Conflicts of Interest

The authors declare no conflicts of interest.

## Data Availability

The data that support the findings of this study are available from the corresponding author upon reasonable request.
